# Comparison of female and male behavior in the elevated gradient of aversion

**DOI:** 10.1590/1414-431X2022e11892

**Published:** 2022-02-16

**Authors:** R. Bonuti, S. Morato

**Affiliations:** 1 Laboratório de Comportamento Exploratório, Faculdade de Filosofia, Ciências e Letras de Ribeirão Preto, Universidade de São Paulo, Ribeirão Preto, SP, Brasil

**Keywords:** Anxiety, Exploratory behavior, Impulsivity, Gender differences, Fear, Self-protection

## Abstract

The elevated gradient of aversion (EGA) is an apparatus for investigating the exploratory behavior of rats in 3-min sessions, consisting of three different sections of the same size: tunnel, closed arm, and open arm. Factorial analyses have defined three factors: exploration, impulsivity, and self-protection. In general, male rats are placed in the tunnel end and tend to hesitate leaving this starting point. Then, they hesitate leaving the tunnel and entering the closed arm, which they explore and tend to avoid entering the open arm or even just stick their head in and not enter it at all. Since females were not used for this test and are reported to be more explorative than male rats, the present work aimed to compare the behavior of male and female rats in the EGA. Thirty male and 34 female Wistar rats were submitted to 3-min sessions in the EGA. In general, results indicated that females were different from males: they explored more (Factor 1 - Exploration), are more impulsive (Factor 2 - Impulsivity), and are less anxious/fearful (Factor 3 - Self-protection). These results confirmed the results of other studies obtained with other apparatuses and show that females exhibit higher locomotion than males and are less anxious/fearful.

## Introduction

We have recently reported a new apparatus for scoring rat behavior in a novel environment (1). A factorial analysis indicated rat behavior is grouped into three factors: exploration, impulsivity, and self-protection. The exploration factor included number of entries into the divisions, time spent in the closed arm, and total number of entries in the whole apparatus. The impulsivity factor included latency of the first entry into the open arm, number of entries, and time spent in it. The self-protection factor included measures related to the total time spent in the more protected area. It is not easy to determine the motivations behind these factors, but from the measures discussed, exploration may involve curiosity or information-seeking or simple motor activity. Impulsivity may involve lack of control, while self-protection may involve anxiety and/or fear. These factors were confirmed by comparison with the behavior of rats in other apparatuses (open-field and elevated plus-maze) and with the use of drugs affecting behavior (1).

The above study (1) was conducted only with males. However, it is well known that the behavior of males and females is rather different when tested in the open-field (2-9) or in the elevated plus-maze (10-13). Thus, the aim of the present study was to compare the behavior of males and females submitted to the elevated gradient of aversion (EGA).

## Material and Methods

### Subjects

Thirty male (approximately 220 g) and 34 female (approximately 190 g) 60-day old Wistar rats were obtained from the animal house of the State University of Campinas, Brazil. They were housed in groups of five in polypropylene cages (41×34×17 cm) with rat chow (Nuvilab, Brazil) and tap water *ad libitum*. The animal room was maintained on a 12-h light/dark cycle (lights on at 7:00 a.m.) with temperature kept between 24 and 27°C. Cleaning of the cages was performed three times a week and dust-free wood shavings were used as bedding. All testing was performed between 7:30 and 11:30 a.m. All experimental procedures were carried out in accordance with the Guidelines of the Brazilian Society for Neuroscience and Behavior recommendations for animal care and with the U.K. Animals (Scientific Procedures) Act, 1986, and associated guidelines.

### Apparatus

**Figure 1:**
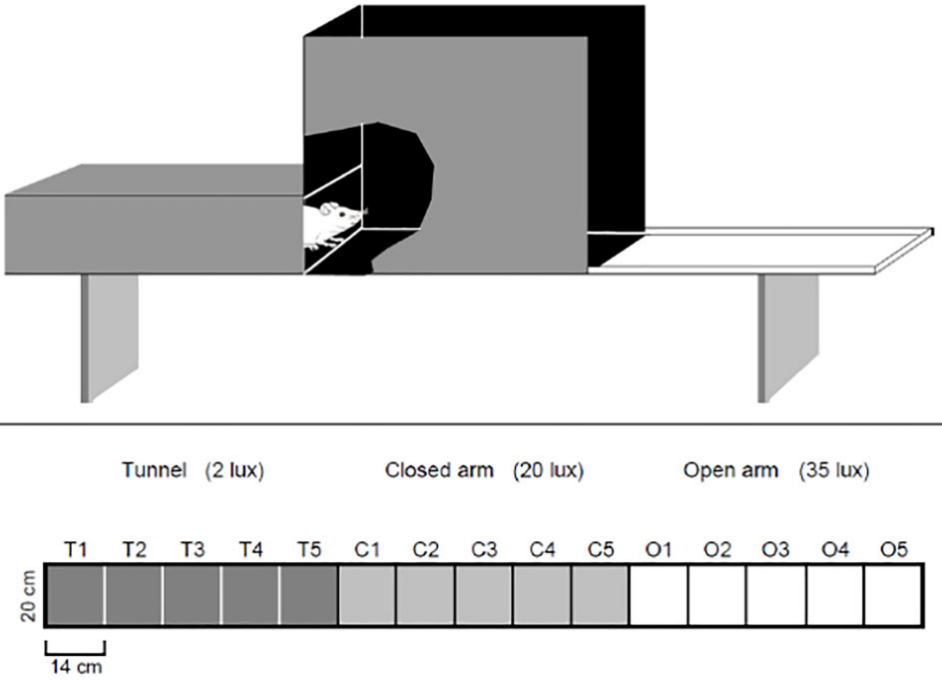
Schematic representation (upper panel) of the elevated gradient of aversion (EGA). Lower panel, top view of the EGA: dark gray indicates the tunnel compartment with area division (T1 to T5), light gray indicates the closed arm compartment (C1 to C5), and white indicates the open arm compartment (O1 to O5).

The apparatus was composed of three compartments equal in size with different motivational properties. It consisted of a 210×20-cm alley divided into three 70×20-cm distinct areas (Figure 1). The first compartment (used as the starting point), at one end of the apparatus, was tunnel-shaped, enclosed with walls 25-cm high, and covered with transparent red Plexiglas (which allows observation and video recording), with the floor and walls lined with black opaque Formica. At the entrance of the tunnel (rectangle T1), there was a sliding door (20×25 cm) through which subjects were introduced into the apparatus, which was the starting point. The other end of the tunnel was connected to the second (middle) compartment, which was surrounded by walls 60-cm high. Both the floor and walls were lined with black opaque Formica and resembled a closed arm of the elevated plus-maze. This second compartment was connected to the third section, which had no walls and was lined with white opaque Formica surrounded by a 1-cm high white rib to prevent rats from falling off the apparatus. This section resembled the open arm of the elevated plus-maze. The whole apparatus was elevated 50 cm above the ground.

### Procedures

Each subject was individually placed at the beginning of the tunnel, then the sliding door was closed and the animal was allowed to move freely for 3 min. After each session, the apparatus was cleaned with a 5% ethanol solution and dried with a cloth. The tests were carried out in a room lit by a 60-W incandescent light bulb 2.7 m above the ground, which provided the following light intensities measured at the center of each compartment: 2 lux inside the tunnel, 20 lux inside the closed arm, and 35 lux in the open arm.

All behavioral tests were recorded using a video camera (Sony, Brazil) placed above the EGA and connected to a video recorder in an adjacent room. The videos were subsequently analyzed by a trained observer. Behavioral parameters were scored with a behavior scoring freeware (X-PloRat) developed at the Laboratory of Exploratory Behavior, University of São Paulo at Ribeirão Preto, Brazil (14). To record the behavior of the subjects, the EGA floor image was divided into fifteen 14×20-cm rectangles on a transparent plastic sheet placed on the computer screen. The number of entries and time spent in each rectangle were used to determine the measures included in each factor. Entry into a rectangle was scored when all four paws of the rat had entered the rectangle.

### Data analysis

Data are reported as means±SE and comparisons between males and females were analyzed by Student's *t*-test. In all cases, the level of significance was set at P<0.05.

## Results

**Figure 2:**
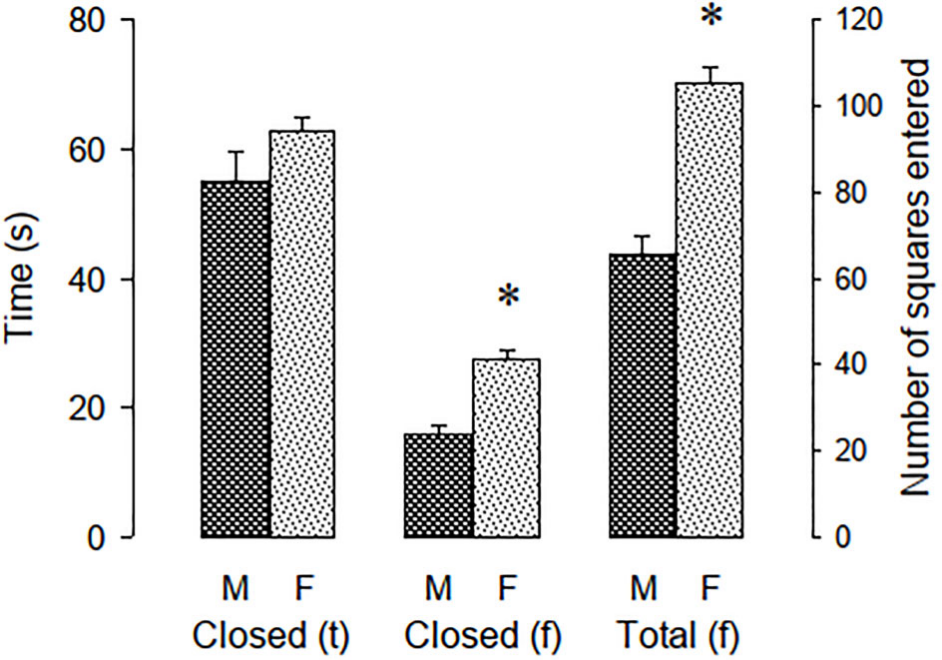
Measures included in the exploration factor. Closed (t): time spent in the closed arm; Closed (f): number of squares entered in the closed arm; Total (f): total number of entries in the elevated gradient of aversion; M: males; F: females. Data are reported as means±SE. *P<0.05 compared to males (unpaired Student's *t*-test).

Figure 2 shows the measures included in factor 1, exploration. Females and males did not differ in time spent in the closed arm (F[1, 62]=2.458, P=0.122). In addition, female rats entered more rectangles in the closed arm than male rats (F[1, 62]=37.961, P<0.001). Finally, female rats entered more rectangles than males overall (F[1, 62]=47.043, P<0.001).

**Figure 3:**
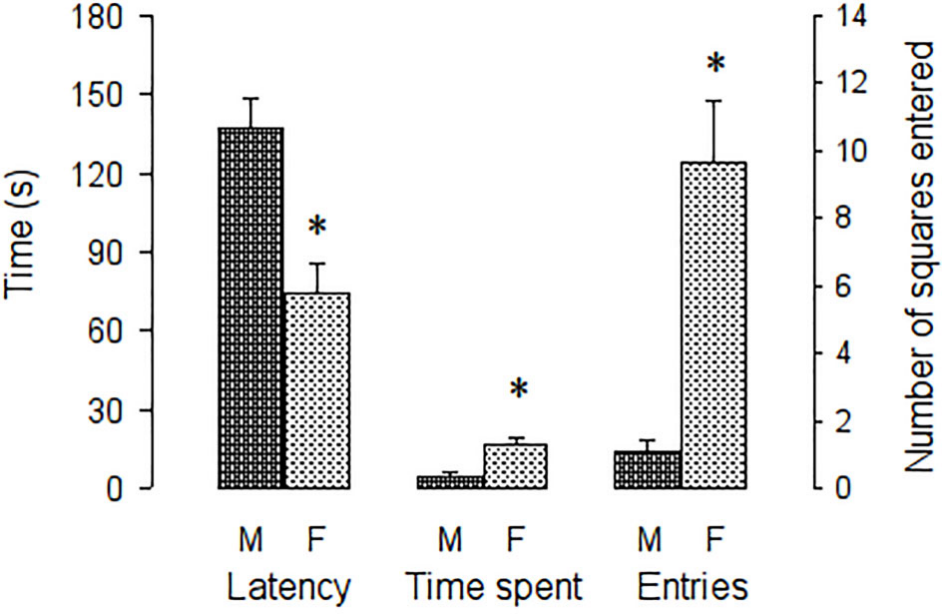
Measures included in the impulsivity factor. Latency of first entry into the open arm, time spent in the open arm, and number of squares entered in the open arm are reported as means±SE. *P<0.05 compared to males (unpaired Student's *t*-test). M: Males; F: females.

Figure 3 shows factor 2 measures, impulsivity. It shows that females exhibited a shorter latency than males to enter the open arm for the first time (F[1, 62]=15.203, P<0.001). Also, the figure shows that females spent more time in the open arm (F[1, 62]=13.086, P<0.001) and entered more rectangles in the open arm than males (F[1, 62]=17.469, P<0.001).

**Figure 4:**
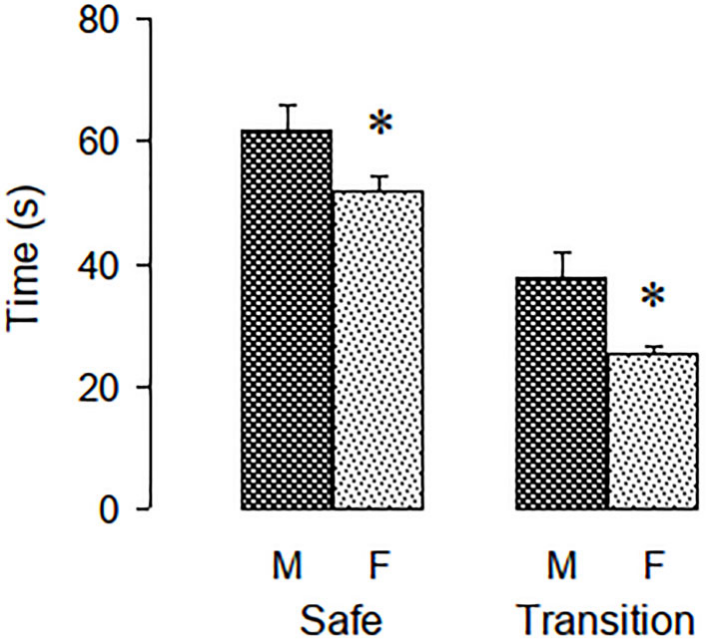
Measures included in the self-protection factor. Safe: time spent in the safe area of the elevated gradient of aversion (EGA) (first and second rectangles of the tunnel); transition: time spent in the transition area of the EGA (fifth rectangle of the tunnel, next to the first rectangle of the closed arm). M: Males; F: females. Data are reported as means±SE *P<0.05 compared to males (unpaired Student's *t*-test).

Figure 4 shows the measures included in factor 3, self-protection. Females spent less time than males in both the safe area - rectangles 1 and 2 (F[1, 62]=4.678, P=0.034) and the transition area - rectangle 5 (F[1, 62]=9.013, P=0.004).

## Discussion

In general, our results showed different behavior between females and males, with females exploring more, as measured by the greater number of rectangles entered in a similar time period. The difference in exploratory behavior (Factor 1) are easily explained by the abundance of reports in the literature indicating that females explore more than males in apparatuses such as the open-field, the elevated plus-maze, and the light/dark box (2-13,15-17). In all of these reports, this type of data is interpreted as decreased fear/anxiety.

In general, females have been found to be more impulsive than males, as defined in Rico et al. (1), that is, taking spontaneous behaviors into account. Females entered the open arm earlier than males, explored it more, and spent more time in it. Possible explanations for this difference are: females have been reported to exhibit less anxiety/fear and higher locomotion (9-12). Similar explanations could be used for the lower scores on self-protection (Factor 3): females left the safe area and the transition area earlier than males. In conclusion, females respond to the EGA, but in a different way than males.

## References

[1] Rico JL, Bonuti R, Morato S (2019). The elevated gradient of aversion: a new apparatus to study the
rat behavior dimensions of exploration, impulsivity and fear. Braz J Med Biol Res.

[2] Broadhurst PL (1958). Determinants of emotionality in the rat: III. Strain
differences. J Comp Physiol Psychol.

[B03] Satinder KP (1968). A note on the correlation between open field and escape avoidance
behavior in the rat. J Psychol.

[B04] Valle FP (1970). Effects of strain, sex, and illumination on open-field behavior
of rats. Am J Psychol.

[B05] Masur J (1972). Sex differences in “emotionality” and behavior of rats in the
open-field. Behav Biol.

[B06] Gray AF, Lalljee B (1974). Sex differences in emotional behavior in the rat: correlation
between open-field defecation and active avoidance. Anim Behav.

[B07] Archer J (1975). Rodent sex differences in emotional and related
behavior. Behav Biol.

[B08] Beatty WW, Fessler RG (1976). Ontogeny of sex differences in open-field behavior and
sensitivity to electric shock in the rat. Physiol Behav.

[9] Slob AK, Huizer T, Van der Werff ten Bosh JJ (1986). Ontogeny of sex differences in open-field ambulation in the
rat. Physiol Behav.

[B10] Johnston AL, File SE (1991). Sex differences in animal tests of anxiety. Physiol Behav.

[B11] Steenbergen HL, Farabollini F, Heinsbroek RP, Van de Poll NE (1991). Sex-dependent effects of aversive stimulation on holeboard and
elevated plus-maze behavior. Behav Brain Res.

[B12] Imhof JT, Coelho ZM, Schmitt ML, Morato GS, Carobrez AP (1993). Influence of gender and age on performance of rats in the
elevated plus-maze apparatus. Behav Brain Res.

[13] Marcondes FK, Miguel KJ, Melo LL, Spadari-Bratfisch RC (2001). Estrous cycle influences the response of female rats in the
elevated plus-maze test. Physiol Behav.

[14] Tejada J, Correio KTC, Morato S (2018). X-PloRat: a software for scoring animal behavior in enclosed
spaces. Psicol Teo Pesqui.

[15] Bourin M, Hascoët M (2003). The mouse light/dark box test. Eur J Pharmacol.

[16] Arrant AE, Schramm-Sapyta NL, Kuhn CM (2013). Use of the light/dark test for anxiety in adult and adolescent
male rats. Behav Brain Res.

[17] Domonkos E, Borbélyová V, Csongová M, Bosý M, Kačmárová M, Ostatníková D (2017). Sex differences and sex hormones in anxiety-like behavior of
aging rats. Horm Behav.

